# High Prevalence of *Giardia duodenalis* Assemblage B Infection and Association with Underweight in Rwandan Children

**DOI:** 10.1371/journal.pntd.0001677

**Published:** 2012-06-12

**Authors:** Ralf Ignatius, Jean Bosco Gahutu, Christian Klotz, Christian Steininger, Cyprien Shyirambere, Michel Lyng, Andre Musemakweri, Toni Aebischer, Peter Martus, Gundel Harms, Frank P. Mockenhaupt

**Affiliations:** 1 Institute of Tropical Medicine and International Health, Charité – University Medicine Berlin, Berlin, Germany; 2 University Teaching Hospital of Butare, Faculty of Medicine, National University of Rwanda, Butare, Rwanda; 3 Robert Koch-Institute, Department of Mycology and Parasitology, Berlin, Germany; 4 Institute of Clinical Epidemiology and Applied Biometry, Eberhard Karls University, Tübingen, Germany; University of Pittsburgh, United States of America

## Abstract

**Background:**

*Giardia duodenalis* is highly endemic in East Africa but its effects on child health, particularly of submicroscopic infections, i.e., those below the threshold of microscopy, and of genetic subgroups (assemblages), are not well understood. We aimed at addressing these questions and at examining epidemiological characteristics of *G. duodenalis* in southern highland Rwanda.

**Methodology/Principal Findings:**

In 583 children <5 years of age from communities and health facilities, intestinal parasites were assessed by triplicate light microscopy and by PCR assays, and *G. duodenalis* assemblages were genotyped. Cluster effects of villages were taken into account in statistical analysis. The prevalence of *G. duodenalis* as detected by microscopy was 19.8% but 60.1% including PCR results. Prevalence differed with residence, increased with age, and was reduced by breastfeeding. In 492 community children without, with submicroscopic and with microscopic infection, underweight (weight-for-age z-score <−2 standard deviations) was observed in 19.7%, 22.1%, and 33.1%, respectively, and clinically assessed severe malnutrition in 4.5%, 9.5%, and 16.7%. Multivariate analysis identified microscopically detectable *G. duodenalis* infection as an independent predictor of underweight and clinically assessed severe malnutrition. Submicroscopic infection showed respective trends. Overall, *G. duodenalis* was not associated with gastrointestinal symptoms but assemblages A parasites (proportion, 13%) were increased among children with vomiting and abdominal pain.

**Conclusions/Significance:**

The prevalence of *G. duodenalis* in high-endemicity areas may be greatly underestimated by light microscopy, particularly when only single stool samples are analysed. Children with submicroscopic infections show limited overt manifestation, but constitute unrecognized reservoirs of transmission. The predominance of assemblage B in Rwanda may be involved in the seemingly unimposing manifestation of *G. duodenalis* infection. However, the association with impaired child growth points to its actual relevance. Longitudinal studies considering abundant submicroscopic infections are needed to clarify the actual contribution of *G. duodenalis* to morbidity in areas of high endemicity.

## Author Summary

*Giardia duodenalis* is a protozoan parasite causing gastroenteritis. Although the parasite occurs worldwide, its regional prevalence varies considerably. Using PCR as a highly sensitive molecular diagnostic tool, we detected *G. duodenalis* in 60% of 583 children younger than five years in southern Rwanda. It was by far the most frequent intestinal parasite detected in this population. Importantly, two out of three infections would have been undetected if only the commonly used light microscopy had been applied. Genotyping revealed the presence of two distinct types of parasites, and only the infrequent subtype showed a weak association with gastrointestinal symptoms. However, *G. duodenalis* infection was associated with underweight and clinically assessed severe malnutrition. The data call for the establishment of more sensitive than light microscopy, yet simple diagnostic tools to identify infected children as well as for the consideration of abundant submicroscopic infections in evaluating the significance of *G. duodenalis* in high endemicity areas.

## Introduction

*Giardia duodenalis* (syn. *G. intestinalis*, *G. lamblia*) is among the most common intestinal protozoa and is the most frequent parasitic agent of gastroenteritis worldwide. The regional prevalences of infection differ enormously and may be >30% in children in the African and the Eastern Mediterranean region [1]. Although *G. duodenalis* is known for causing gastrointestinal symptoms, such as acute or chronic diarrhoea, bloating, and stomach cramps, asymptomatic infections may occur, particularly in highly endemic areas, and there is also evidence for protection against acute diarrhoea in infected individuals [2]–[4]. Chronic (or recurrent) infection has been associated with malnutrition, wasting and stunting, most likely due to malabsorption caused by the parasites, and with reduced cognitive functions at later age [5]. The pathogenetic determinants are poorly understood but may involve both host and parasite factors [2]. As for the latter, eight major genetic groups of *G. duodenalis* have been revealed, i.e., assemblages A1, A2, and B–H with A1, A2, and B being considered pathogenic in humans [6], [7]. Assemblages have inconsistently been linked with symptoms: assemblage A parasites have been associated with more severe clinical symptoms as compared to assemblage B parasites in Australia, Bangladesh, Peru, Spain, and Great Britain [8]–[13], but the opposite has been reported from the Netherlands and Ethiopia [14], [15], and no association in Brazil [16]. Assemblage-associated differences in the pathogenesis of giardiasis have been observed in murine studies where the infection with clone GS (assemblage B), but no with clone WB (assemblage A), caused disaccharide deficiency in infected animals [17].

Diagnosis of *G. duodenalis* is traditionally based on the detection of the parasites by light microscopy in direct stool smears or following concentration techniques, e.g., by formalin-ethyl acetate centrifugation. Multiple rather than single sample testing is recommended to improve sensitivity but this is often difficult to implement. Immunoassays are of superior sensitivity but also more expensive [18], [19]. Similarly, highly sensitive PCR-based methods have been developed but rarely applied in developing countries so far [20]. Limited evidence shows that prevalence estimates based on PCR may exceed those derived by microscopy, e.g., recently in western Uganda where up to two thirds of people in rural areas were found to be (asymptomatically) infected [21]. No data are available, however, regarding the epidemiology and clinical significance of submicroscopic *G. duodenalis* infections, i.e., infections detected by PCR but not by light microscopy, in highly endemic areas.

East Africa is considered to be among the most endemic regions for *G. duodenalis* surpassed only by the Indian subcontinent [22] although recent community-based figures are rare. In the present study from southern Rwanda, we aimed at assessing prevalences and epidemiological features of *G. duodenalis* infection among children in communities and health facilities applying both microscopy and PCR for diagnosis. Also, we aimed at examining the influence of *G. duodenalis* infection, particularly of submicroscopic infection and different assemblages, on the children's clinical status.

## Methods

### Ethics statement

All children's parents were thoroughly informed on the purpose and procedures of the study, and recruitment was preceded by HIV pre-counseling and obtaining informed written consent from the children's parents. The study was reviewed and approved by the National Ethics Committee, Republic of Rwanda.

### Study area and sampling

The study was conducted from January to March 2010, i.e. during a delayed rainy season, in Butare and the neighbouring rural Huye subdistrict. Butare (population approximately 100,000; altitude 1,768 m) is the capital of Huye district, southern province of Rwanda. Located on the country's central plateau (average altitude, 1,700 m; yearly rainfall, 1,200 mm; mean temperature, 19°C), the city is surrounded by densely populated farmland hills. Governmental health services in the area are provided by several primary health centres, Kabutare district hospital and the University Teaching Hospital of Butare (CHUB, *Centre Hospitalier Universitaire de Butare*). Due to Rwanda's mandatory health insurance system, treatment of common diseases is basically free of charge including utilization of district and provincial hospitals provided there is adherence to a strict referral system [23].

**Figure 1:**
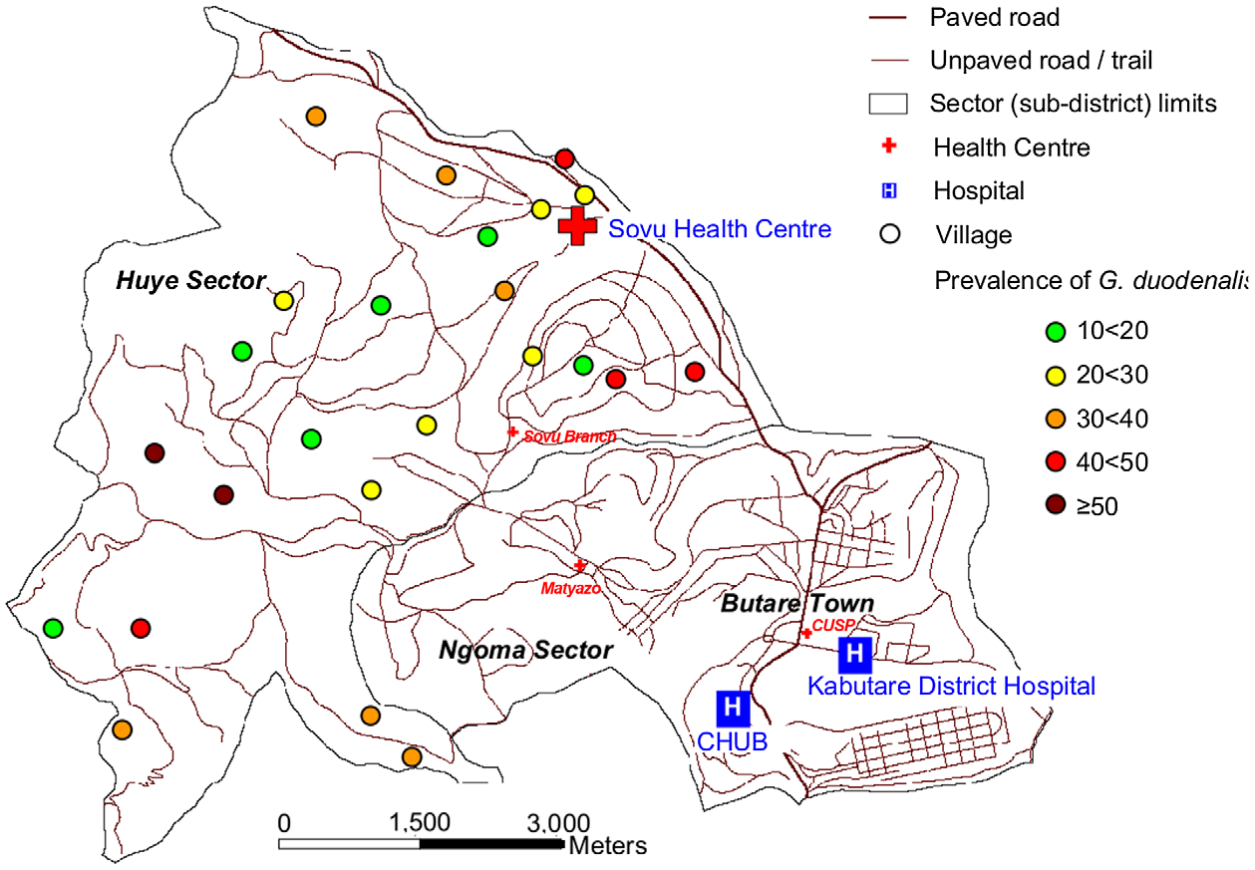
Distribution of *Giardia duodenalis* in children <5 years of age in Huye sector, Rwanda.

The study was designed as a cross-sectional survey to assess the prevalences of malaria, HIV, and intestinal pathogens in children under five years of age in the CHUB catchment area, i.e., at the levels of community, health center, and district hospital. The present report focuses on *G. duodenalis*. Details of the study among a total of 749 children have been described previously [24]. Briefly, for the community level and based on most recent census data, each 25 households were randomly chosen by bottle-spin in a total of 24 randomly selected villages (out of 45) in the rural Huye subdistrict (population approximately 20,000; Figure 1). Community health workers randomly selected one child per family, and asked the child to be presented to the study team located at Sovu health center (or a non-permanently staffed branch) on a scheduled (usually next) day (*n* = 545). In parallel and during 16 and 11 days, respectively, 103 and 101 pediatric health facility attendants aged five years or less and presenting with a health problem at the primary Sovu health center and at the referral Kabutare district hospital, respectively, i.e. the health facilities serving this population, were consecutively recruited. Leading primary diagnoses in the health facility attendants were respiratory tract infection, gastro-intestinal tract affection, and malaria. Clinical details are reported elsewhere [24].

### Sample collection and examinations

Brief questionnaires were filled in on socio-economic aspects of the children's families including household assets. Age, sex, weight, and fever (axillary temperature ≥37.5°C) were documented. Breastfeeding was documented as any *vs.* none. Using standardized forms, children were examined by a physician and a medical history was obtained. For the latter, parents were specifically asked about the presence of gastrointestinal and other symptoms during the preceding two weeks, e.g., vomiting, diarrhoea, and abdominal pain. For this study, we used two categories to describe impaired child growth and/or malnutrition, i.e. underweight and clinically assessed severe malnutrition. Underweight was defined as a weight-for-age z (WAZ)-score of <−2 standard deviations (SD) following calculation of WAZ-scores using WHO Anthro [25]. Clinically assessed severe malnutrition was evaluated by the study physician (CSh) based on clinical signs, growth percentiles and/or mid-upper-arm circumference. Malaria [24], urinary tract infection (Multistix 10SG, Bayer, Germany), and other diseases were treated according to Rwanda health authority guidelines [26].

In the communities, stool collection containers were handed to the parents who were instructed to bring a fresh sample of the selected child at the scheduled day of examination. In the health facilities, fresh samples were collected. During presentation of the children, intestinal pathogens were screened for by direct wet mount microscopic stool examination. A second microscopic examination was performed later on the examination day following the ether-based concentration technique [27]. Aliquots of stool were conserved in merthiolate-iodine-formaldehyde (MIF), transported to Berlin and once more examined microscopically after ether-based concentration. Triplicate microscopy of identical samples was performed to identify potential flaws in the diagnostic procedures and to reduce inter-observer variability. Of the 749 children, 86,1% (645) provided a stool sample: 95.4% of the community children (520/545) but only 61.3% (125/204) of health facility attendants. Overall, 622 stool samples could be assessed by all three microscopic examinations. Quantification of pathogens was omitted. Samples were considered microscopically positive for a given parasite if it was detected in at least one of the three assays, and negative if negative in all three assays.

For molecular assays, aliquots of stool were stored at −70°C and transported on dry ice to Berlin. Of the 622 microscopically assessed samples, 583 were available for PCR analysis. DNA was extracted by commercial kits (Qiamp DNA Stool Mini Kit, Qiagen). For each sample, phocine herpesvirus 1 (PhHV-1), kindly provided by Dr. Martin Schutten, Department of Virology, Erasmus MC, Rotterdam, The Netherlands, was added to the isolation lysis buffer to serve as an internal control for the extraction process [28]. Infections with *Necator americanus*, *Ancylostoma duodenalis*, or *Ascaris lumbricoides* were detected by real-time PCR assays [29], [30]. A multiplex real-time PCR assay with pathogen-specific fluorescent detection probes was performed to identify *G. duodenalis*, *Entamoeba histolytica* (or *E. dispar*), and *Cryptosporidium parvum* [20]. Real-time PCR assays were run on a Lightcycler 480 (Roche Diagnostics) including controls from microscopically confirmed, positive *G. duodenalis* samples as well as negative controls. Cycle threshold (Ct) values of >36 were considered to reflect limited reproducibility due to low copy numbers, and all respective assays were repeated. For *G. duodenalis*, the real-time PCR was repeated for 62 samples with an initial Ct-value of 36.01–45.1 (median, 37.7), of which 47 were reproducibly positive (Ct-value, range, 21.6–38.3; median, 35.0) while 15 were negative. No sample had to be excluded because of evidence of fecal inhibitory factors (Ct-value for the internal control PhHV-1 >36).

### Typing of *G. duodenalis* isolates

For *G. duodenalis* genotyping, stool DNA samples were analysed by a previously described method with slight modifications [31]. A DNA fragment of the *Giardia* triosephosphate isomerase (TPI) gene was amplified by nested PCR using primer TPI-f1, AAATYATGCCTGCTCGTCG and TPI-r1, CAAACCTTYTCCGCAAACC for the first PCR reaction and primer TPI-f2, CCCTTCATCGGNGGTAACTT and TPI-r2 GTGGCCACCACNCCCGTGCC for the second PCR reaction. The PCR products were purified (QIAquick, Quiagen, Hilden, Germany) and unidirectional sequencing reactions were performed with the ABI Big dye 3.1 Terminator kit using TPI-f2 primer and send for in-house sequencing service at the Robert Koch-Institute. Sequences were analyzed with Geneious Pro software tool (Biomatters Ltd., Auckland, New Zealand). To determine *Giardia* genotypes, a sequence fragment from base 610 to 1052 of a reference sequence (Genbank accession number L02120) was compared to previously defined TPI references [32]. The further subdivision of assemblage B isolates into subtypes based on analyzing the *tpi* locus was not attempted because of the known heterozygosity at this locus [32], which we also observed in the majority of isolates typed here.

### Statistical analysis

Complete data sets were available for 583 children. Data analysis was performed using SPSS for Windows (release 18) and SAS for Windows (release 9.2) (SAS Institute Inc.). Descriptive analyses include absolute frequencies and percentages or means, medians, and ranges. Evaluation of determinants of *G. duodenalis* infection and of clinical symptoms was performed by simple and multiple logistic regression analysis, and odds ratios were reported. Confirmatory analyses (means, percentages, *P*-values, significance tests, 95% confidence intervals (95% CIs)) were adjusted for intracluster correlations (cluster = villages) by use of Generalized Estimating Equations (GEE; [33], exchangeable correlation structure for subjects within identical villages). Of note, this leads to discrepancies between “naively” computed percentages from absolute frequencies and reported percentages. Therefore, prevalences reported herein are weighed with respect to differing cluster sizes and do not necessarily correspond to the additionally given absolute numbers. A *P*-value≤0.05 was considered statistically significant.

## Results

Socio-demographic and selected clinical characteristics of the 583 children are shown in Table 1. The majority of children was recruited from the community (84%) and of rural residence. Most parents were farmers or laborers, and many had no formal education. The monthly family income was low as was household asset ownership. Further characteristics have been described elsewhere [24].

**Table 1:** Characteristics of 583 children from southern Rwanda.

Parameter	Value
No.	583
Age (months; median, range)	32.0 (0.5–60)
Female sex (%)	46.2 (270/582)
Breastfed (%)	50.4 (298/582)
Recruited in community (%)	84.4
Rural residence (%)	90.3 (524/578)
Household income<median (5000 RwF)	30.3 (207/582)
Health insurance present (%)	37.6 (291/582)
Lacking formal education, mother (%)	26.9 (162/581)
Mother's occupation: farmer/laborer (%)	97.3 (565/580)
Lacking formal education, father (%)	29.8 (190/571)
Father's occupation: farmer/laborer (%)	82.5 (485/579)
No. of people/household (median, range)^a^	5.0 (2–12)
No. of siblings (median, range)^a^	2.0 (0–9)
Absence of assessed household assets (%)	42.3 (242/581)
History of fever (48 hours, %)	29.9 (92/541)
Febrile (%)	9.4 (46/581)
Malaria (%)	4.6 (25/583)
HIV positive (%)	1.2 (7/583)

Cluster effects are taken into account.

a , *n* = 582.

### Prevalence of intestinal parasites

Gastrointestinal parasites were assessed in all children (Table 2). *G. duodenalis* was the most prevalent parasite identified, detected by microscopy in 19.8% (114/583) and by PCR in 59.7% (366/583) (Table 2). All but two of the microscopically positive samples were also detected by PCR (i.e., total positivity, 60.1% (368/583)). In 51.3% (254/469) of samples diagnosed as negative for *G. duodenalis* by microscopy, PCR yielded a positive result. Setting *G. duodenalis* detected by any means as reference, the sensitivity of microscopy in detecting the parasite was 30.8% (95%CI, 25.3–36.8%) and that of PCR was 99.5% (95%CI, 97.9–99.9%).

**Table 2:** Prevalence of intestinal parasites in 583 children from southern Rwanda.

Parasite	Prevalence (%)
*Giardia duodenalis* a	60.1
Microscopic	19.8
Submicroscopic	40.1
*Ascaris lumbricoides* a	31.6
*Cryptosporidium parvum*	4.9
*Necator americanus* b	2.7
*Entamoeba histolytica*	1.1
*Trichuris trichiura*	1.9
*Strongyloides stercoralis*	0.7
*Balantidium coli*	0.2
*Entamoeba coli*	27.6
*Entamoeba dispar*	14.7
*Blastocystis hominis*	4.1
*Iodamoeba bütschlii*	3.4
*Trichomonas hominis*	3.3
*Endolimax nana*	3.0
*Chilomastix mesnili*	2.4

Prevalence estimates take into account cluster effects.

a , includes samples positive by microscopy only;

b , includes 3/16 microscopically detected hookworms without PCR confirmation and species differentiation.

Of 208 successfully typed isolates, 85.9% (179/208) were assemblage B and 12.7% (27/208) were assemblage A2, in addition to one assemblage A1 and one mixed assemblage A+B. Infections with assemblage B isolates were more frequently submicroscopic (53.2%, 95/179) than infections caused by assemblage A (28.0%, 7/28; *P* = 0.02).

*A. lumbricoides* was the second most common intestinal pathogen affecting almost one third of the children. Other intestinal pathogens were comparatively rare (Table 2). The presence of *G. duodenalis* was positively associated with the apathogenic species *E. coli*, *E. dispar*, and *I. bütschlii*, and negatively with *C. parvum* and *E. histolytica* (each, *P*<0.05). Overall, 18.0%, 33.3%, 25.9%, 13.2%, and 9.6% of the children harbored 0, 1, 2, 3 or ≥4, respectively, of the parasites listed in Table 2. The most common constellations were *G. duodenalis* mono infections (22.1%), followed by co-infestations with *G. duodenalis* and *A. lumbricoides* (9.8%) or with *E. coli* (5.7%), and triple infestations with *G. duodenalis*, *A. lumbricoides*, and *E. coli* (3.9%). Assemblages A and B did not associate with any other intestinal parasite species (data not shown).

Table S1 displays the distribution of intestinal parasites separated for community children and health facility attendants.

### Factors associated with *Giardia duodenalis* infection

Factors potentially associated with the presence of *G. duodenalis* were analyzed including available socio-economic data. *G. duodenalis* was more common in community children (65.2%) than in health facility attendants (46.4%; *P* = 0.001) (Table 3), and varied with residence (*P* = 0.002). Among community children, prevalences in the villages ranged from 26.7% to 87.0% (*P*<0.0001) but no geographic focus was discernible (Figure 1). Moreover, age had a prominent impact in community children: the prevalence of *G. duodenalis* increased from 34.1% (14/44) in infants to 81.1% (79/90) in children ≥4 years of age (*P*<0.0001). This was not observed in health facility attendants (*P* = 0.10). Also, the proportion of microscopic among all *G. duodenalis* infections in community children tended to increase with age. While only 7.4% (1/15) of infections in infants were microscopically detectable, this was the case in 30.8% (21/68), 30.3% (26/88), 43.3% (36/82), and 29.3% (21/73), in the age groups 1<2, 2<3, 3<4, and 4<5 years, respectively (*P* = 0.06).

**Table 3:** Univariate and multivariate analysis of factors associated with *Giardia duodenalis*.

Parameter	n	*G. duodenalis* positive (%)	OR	95%CI	*P*	aOR ^a^	95%CI	*P*
Subgroup								
Community	492	65.2	1					
Health facility	91	46.4	0.46	0.29–0.73	0.001	0.46	0.27–0.78	0.004
Age (years)								
<1	61	32.7	1					
1<2	141	48.9	1.97	1.08–3.59	0.03	1.66	0.90–3.04	0.10
2<3	144	64.9	3.82	2.06–7.07	<0.0001	2.72	1.41–5.26	0.003
3<4	124	68.7	4.52	2.65–7.72	<0.0001	2.55	1.19–5.45	0.02
4<5	113	72.8	5.52	3.15–9.70	<0.0001	3.34	1.47–7.50	0.004
Breastfed								
No	284	71.5	1					
Yes	298	50.2	0.40	0.32–0.51	<0.0001	0.62	0.40–0.97	0.04
No. of siblings								
None	134	52.4	1					
1–3	406	61.6	1.45	0.99–2.12	0.05	1.15	0.74–1.80	0.54
≥4	42	77.6	3.14	1.87–5.27	0.0001	2.47	1.19–5.12	0.02

OR, odds ratio; 95%CI, 95% confidence interval, aOR ^a^, adjusted odds ratio, adjusted for all other factors shown in table and taking into account cluster effects (*n* = 576; R^2^ = 0.24).

Interestingly, breastfeeding reduced the odds of being infected with *G. duodenalis* (*P* = 0.04) while a high number of siblings showed the opposite effect. In multivariate analysis, *G. duodenalis* remained positively associated with increasing age many siblings, and negatively with health facility attendance and breastfeeding (Table 3). Separating into microscopic and submicroscopic infections among community children, only age remained significantly associated in each case (data not shown).

Several factors expected to be associated with *G. duodenalis* were actually not, including parents' education, household income, household assets, the number of individuals living in the household, availability of piped water, and household cattle possession (data not shown). Only 11 children (1.8%) had reportedly taken drugs with anti-giardial effects within the preceding two weeks (metronidazole, 2; mebendazole, 9). In these, *G. duodenalis* was found in 45.5% (5/11) as compared to 60.5% (361/581) of respectively untreated children (*P* = 0.27).

For assemblages B and A, no differences with respect to the above mentioned factors were discernible. In particular, similar proportions of *G. duodenalis* were of assemblage A in community children (14.3%, 25/186) and health facility attendants (16.0%, 3/21; *P* = 0.99), and in households with (22.0%, 6/35) and without cattle possession (13.5%, 22/171; *P* = 0.50). The age (median months, range) of infected children with assemblage A or assemblage B parasites did not differ (38.0 (22–59) *vs.* 37.0 (8–60), *P* = 0.24). A notable exception was residence: in the community children's villages, the proportion of assemblage A among detected *G. duodenalis* ranged between 0% and 50% (*P* = 0.02) but again no focus was discernible.

### Clinical significance of *Giardia duodenalis* infection in community children

We focused the analysis of the clinical significance of *G. duodenalis* infection on community children because of their large sample size and because of the diverse additional morbidity of health facility children [24]. Most community children were asymptomatic: fever and a history of fever were recorded in 3.5% (17/490) and 8.5% (41/452), respectively, and malaria in 2.6% (13/492). Also, gastrointestinal symptoms were rare. Frequencies of loss of appetite, diarrhoea, vomiting, and abdominal pain did not differ between children with and without *G. duodenalis* (Table 4). Defining giardiasis as at least one of the before mentioned gastrointestinal symptoms in the presence of *G. duodenalis*, 69 of 492 community children were affected (based on microscopic diagnosis only, 21 of 492). Abdominal distension tended to be increased in infected children [OR, 2.94 (95%CI, 0.94–9.20), *P* = 0.07].

**Table 4:** Selected clinical characteristics of 492 community children with or without *Giardia duodenalis* infection.

	Uninfected	Infected			Assemblage	
		all	submicroscopic	microscopic	B	A
No.	166	326	221	105	161	25
% febrile	3.4	3.4 (11/324)	2.9 (6/220)	4.7 (5/104)	3.1 (5/160)	8.7
% loss of appetite (history)	10.5	12.0	11.5	13.5	11.4	4.0
% diarrhoea (history)	5.9	6.8	8.1	3.9	7.4	4.2
% vomiting (history)	2.2	2.2	2.9	1.0	0.7	9.0* ^,^ †
% abdominal pain (history)	3.6	4.8	4.8	4.7	3.1	12.0* ^,^ †
% abdominal distension (clinically)	1.8 (3/163)	5.6 (20/322)	5.9 (15/217)*	4.7	7.1 (12/160)*	0
% severe malnutrition (clinically)	4.5 (7/164)	11.6 (46/322)*	9.5 (26/218)*	16.7 (20/104)*	15.9 (28/159)*	9.4 (2/24)
% underweight (WAZ <−2 SD)	19.7 (33/165)	25.5 (84/325)	22.1 (50/221)	33.1 (34/104)*	29.4 (47/160)*	15.6
Median WAZ score (SD; range)	−1.06 (−5.34–2.58)	−1.25 (−5.0–3.26)	−1.15 (−4.97–2.58)	−1.46 (−5.0–3.26)*	−1.34 (−5.0–3.26)*	−1.29 (−4.28–1.0)

Prevalence estimates take into account cluster effects.

*: , *P*<0.05 as compared to uninfected children;

**†:** , *P*<0.05 as compared to children infected with assemblage B parasites.

Underweight (WAZ score <−2 SD) was observed in 23.7% (117/490) of community children. Of note, *G. duodenalis* infected children more frequently exhibited clinically assessed severe malnutrition [OR, 2.15 (1.39–3.35), *P* = 0.0006] than uninfected children. This was paralleled by slightly decreased WAZ scores (*P* = 0.14) and an increased proportion with underweight (*P* = 0.14) among infected children (Table 4).

Looking separately at submicroscopic and microscopic infections, children with microscopic infections exhibited significantly increased odds of clinically assessed severe malnutrition [OR, 3.71 (2.06–6.70), *P*<0.0001] and of underweight [OR, 1.93 (1.23–3.04), *P* = 0.004] as well as reduced WAZ-scores (*P* = 0.01), as compared to uninfected children. Children with submicroscopic infections had an intermediate prevalence of clinically assessed malnutrition [OR, 1.80 (1.14–2.86), *P* = 0.01] but WAZ scores were not significantly reduced (*P* = 0.47).

In multivariate analysis, *G. duodenalis* was independently associated with increased odds of clinically assessed severe malnutrition [adjusted OR (aOR), 2.06 (95%CI, 1.26–3.39); *P* = 0.005], adjusted for lacking paternal education [aOR, 2.57 (1.21–5.46)], and number of siblings [aOR, 1.17 (1.02–1.34)]. All other covariates (age, absence of any household possession, sex, maternal education, household income, possession of cattle, number of additional intestinal parasites) were not significant. In this model, the adjusted odds of clinically assessed severe malnutrition in microscopic *G. duodenalis* infection was 3.34 (1.85–6.42, *P*<0.0001) while submicroscopic infection showed a smaller effect [aOR, 1.76 (1.00–3.09), *P* = 0.05]. An identical approach identified microscopic *G. duodenalis* infection to be an independent predictor of underweight [aOR, 1.83 (1.12–2.99), *P* = 0.02] but not submicroscopic infection [aOR, 1.09 (0.66–1.79)] or *G. duodenalis* infection *per se* [aOR, 1.32 (0.84–2.08)], adjusted for maternal education, and absent household possessions.

Lastly, we examined the clinical relevance of assemblages A and B (Table 4). The increases in underweight and clinically assessed malnutrition observed for *G. duodenalis* infection *per se* were pronounced for assemblage B but less obvious for the small group of children infected with assemblage A parasites. The latter, however, were more frequently affected by abdominal pain (*P* = 0.02) and vomiting (*P* = 0.03) than uninfected children. This was not the case for children with assemblage B isolates. Compared to them, children with assemblage A parasites more frequently reported abdominal pain (*P* = 0.004) and vomiting (*P* = 0.04).

## Discussion

In rural communities in southern highland Rwanda, *G. duodenalis* infects two of three predominantly asymptomatic children, is underestimated by conventional microscopy of single stool samples, and contributes to underweight and clinically assessed malnutrition. This suggests an underrated but considerable burden of disease due to *G. duodenalis*.

The prevalence of *G. duodenalis* of 60% in Rwandan children – who mainly were randomly selected from communities – considerably exceeds figures reported from other parts of the world [1]. The superior sensitivity of PCR in detecting *G. duodenalis* has recently been shown in Danish patients [34]. In contrast, PCR and microscopy had similar sensitivities in a Dutch study [35]. In our study, light microscopy, which commonly is the only method available in resource-poor areas, failed to detect more than two-thirds of actually present *G. duodenalis* infections. Repeated microscopic examinations (which are difficult to implement) or the use of immunoassays could have reduced this discrepancy. In addition, chronically and/or repeatedly infected individuals in highly endemic settings might shed low cyst numbers. Thus, microscopy-based prevalence figures from high-endemicity areas may be underestimations, and analyses comparing individuals categorized by microscopy into *Giardia* infected and non-infected may be confounded by a significant number of false negatives. While some false negatives may persist even when applying PCR assays, that risk cannot be determined accurately in the absence of a well defined gold standard.

As to the clinical significance of *G. duodenalis* among community children, we herein describe associations of symptoms with the presence of the parasite. *G. duodenalis* infection is known to vary widely in clinical manifestation including acute, chronic, and asymptomatic courses [2]. Therefore, we did not attempt to *a priori* define clinical giardiasis in our study. Nevertheless, no evidence for causation of gastrointestinal symptoms was seen, with the potential exception of abdominal distension. The association with malnutrition, however, was impressive: approximately every third and every sixth child with microscopic infection had underweight and clinically assessed severe malnutrition, respectively, although causation cannot be clarified in a cross-sectional study. Association does not mean causality, and reduced sample sizes in subgroup analyses increases the chance of type 1 statistical errors. Selection bias during recruitment at home, e.g., due to preferential presentation by the parents of children with (a recent history of) sickness cannot completely be excluded. However, recruitment teams were instructed to select children from households randomly. Also, confounding cannot completely be excluded but our results remained stable in multivariate analyses adjusting for socio-economic parameters and for the number of other intestinal pathogens serving as a proxy parameter for polyparasitism (data not shown). Notably in this regard, *A. lumbricoides* infections found in one third of the children had no clear-cut effect on malnutrition (data not shown), similar to findings from Brazil and Iran [36], [37]. Clinically assessed severe malnutrition in this study reflects the subjective evaluation of children's status which, nevertheless, was supported by comparison with growth percentiles und MUAC. In interpreting our results, it consequently needs to be kept in mind that this parameter is not standardized. Though, the stronger association with *G. duodenalis* of clinically assessed severe malnutrition as compared to underweight may also indicate a greater selectivity of the former. Unfortunately, we were unable to analyze the influence of *G. duodenalis* on stunting and wasting, i.e., categories based on children's height, because this parameter was inconsistently measured in the field study, had implausible values, and respective Z-scored were consequently omitted. However, our interpretation of malnutrition as a consequence of giardiasis is in line with other studies [37]–[42].

Children with submicroscopic *G. duodenalis* infection had a prevalence of clinically assessed severe malnutrition, which was intermediate between children without and with microscopic infection. In multivariate analysis, submicroscopic infections still tended to be associated with clinically assessed severe malnutrition but showed no link with underweight. Longitudinal observations and immunological studies may help to understand the relevance of this type of infection and the underlying mechanisms.

*G. duodenalis* assemblage B parasites clearly predominated (86%), which is in accordance with most larger studies (recently reviewed in [6]). Interestingly, we detected only one co-infection by both assemblage A and B parasites. By random distribution, a much higher proportion would have been expected possibly reflecting cross-assemblage competition or protection. Unfortunately, we were unable to type all *Giardia* infections. Although the typing rate was higher than in other studies (e.g., [21]) this is most likely due to the fact that typing relied on the single copy *tpi* gene while diagnosis was based on the amplification of the multicopy rDNA. However, this is unlikely to have resulted in a significant bias in the determination of the prevalence of A and B genotypes in the study population. The success of typing correlated with the ct values in the diagnostic PCR, i.e. the content of target DNA in the samples and this was not significantly different for typed samples independent of their A or B status (not shown).

In our study, children infected with assemblage A isolates showed increased proportions of abdominal pain and vomiting while assemblage B infections associated with underweight and clinically assessed severe malnutrition. Much of the available data on the role of assemblages in clinical disease is inconsistent [8]–[16]. Our data are in line, however, with findings from Western Australia where assemblage A parasites were more likely to be found in symptomatic children with diarrhoea while assemblage B parasites were more prevalent in asymptomatic children [8]. Likewise, against high prevalences of assemblage B parasites in Bangladesh, assemblage A isolates were found to be associated with symptomatic disease, i.e., diarrhoea [9], [10]. In contrast, abdominal pain in children was associated with assemblage B parasites in Argentina [43]. Further investigations in our study population will help elucidating whether - in contrast to possibly endemic assemblage B parasites - the genetically distinct assemblage A parasites may appear epidemically and thus are more correlated with acute abdominal symptoms.

Socio-economic factors had no great influence on the presence of *G. duodenalis* in the present study. One reason involved may be a limited selectivity of the questionnaires used in this generally poor agricultural and rural population. Also, we were unable to identify hot spots, let alone their characteristics, potentially underlying the pronounced spatial differences in infection prevalence. In multivariate analysis, only the increases of *G. duodenalis* with children's age and with a high number of siblings as well as the reduction with breastfeeding remained statistically significant. Increasing prevalence with increasing number of siblings supports the role of transmission within households. Protection from *G. duodenalis* infection by breast milk has previously been reported [44]. Considering the high endemicity of *G. duodenalis* in the study area it seems plausible that protective antibodies [45] are transmitted by the breast-milk of seropositive mothers. Soluble IgA has also been shown to contribute to the clearance of *Giardia* spp. in murine studies [46]. In addition, lactoferrin and leukocytes in breast milk could be involved in protection [47], [48].

The association with impaired child growth as observed in the present and other studies [5], [37]–[42] suggests control of *G. duodenalis* infection to be potentially rewarding, e.g. by preventive chemotherapy. Yet, the influence of repeated treatment of *G. duodenalis* infection on anthropometric development is unclear [49]: evidence for a beneficial impact has been observed in Brazilian children [50] but not in Bangladeshi infants [51]. Beyond that, *G. duodenalis* infection in highly endemic areas has been associated with protection from acute diarrhea [3], [4]. Considering the importance of diarrhoea as a leading cause of childhood mortality, such a protective effect might outweigh impaired child growth due to *G. duodenalis* infection. To date, there is insufficient data to balance the potential effects of anti-*Giardia* treatment against each other. Lastly, food supplementation in areas highly endemic for *G. duodenalis* also appears to be complex. *G. duodenalis* associated protection from diarrhea has recently been reported to be lost in Tanzanian children with multi-nutrient supplementation [4] while vitamin A plus zinc supplementation reduced the incidence of giardiasis in Mexican children [52]. Thus, large-scale longitudinal studies are needed to disentangle the role of *G. duodenalis* among the interacting factors contributing to malnutrition and diarrhea in high-endemicity regions and to estimate the potential impact of control measures.

In conclusion, our data provide evidence of a very high prevalence of *G. duodenalis* assemblage B without causing diarrhoea but associated with underweight and clinically assessed severe malnutrition in Rwandan children. The underestimation of *G. duodenalis* by light microscopy suggests that prevalences and consequences have previously been underrated and asks for the implementation of more sensitive diagnostic, yet simple diagnostic tools. The clarification of the clinical significance of *G. duodenalis* in high endemicity areas needs to take account of an abundance of submicroscopic infections.

## Supporting Information

Table S1 **Intestinal parasites in community children and children attending health facilities in southern Rwanda.**
(DOC)

Checklist S1 **STROBE checklist.**
(DOC)
