# Supplementary material for: High Prevalence of Giardia duodenalis Assemblage B Infection and Association with Underweight in Rwandan Children
Source: PLoS Negl Trop Dis. 2012 Jun 12;6(6):e1677. doi: 10.1371/journal.pntd.0001677 (PMC3373622; doi:10.1371/journal.pntd.0001677)
Supplement: Table S1 — Intestinal parasites in community children and children attending health facilities in southern Rwanda. (DOC) [file pntd.0001677.s001.doc]

Table S1. Intestinal parasites in community children and children attending health facilities in southern Rwanda

| Parasite | |  | Community children (%)  *n* = 492 | Health facility patients (%)  *n* = 91 | P |
| --- | --- | --- | --- | --- | --- |
| Giardia duodenalis ^a^ | |  | 66.3 (326) | 46.2 (42) | 0.0003 |
|  | Microscopic | | 21.3 (105) | 9.9 (9) | 0.01 |
|  | Submicroscopic | | 44.9 (221) | 36.3 (33) | 0.13 |
|  | Assemblage A | | 13.4 (25/186) | 14.3 (3/18) |  |
|  | Assemblage B ^b^ | | 86.6 (161/186) | 85.7 (18/21) | 0.91 |
| Ascaris lumbricoides ^a^ | |  | 33.3 (164) | 23.1 (21) | 0.05 |
| Cryptosporidium parvum | |  | 4.5 (22) | 8.8 (8) | 0.09 |
| Necator americanus ^c^ | |  | 2.4 (12) | 4.4 (4) | 0.30 |
| Entamoeba histolytica | |  | 1.2 (6) | 1.1 (1) | 1.0 |
| Trichuris trichiura | |  | 1.6 (8) | 3.3 (3) | 0.39 |
| Strongyloides stercoralis | |  | 0.8 (4) | 0 | 1.0 |
| Balantidium coli | |  | 0.2 (1) | 0 | 1.0 |
|  | |  |  |  |  |
| Entamoeba coli | |  | 30.1 (148) | 16.5 (15) | 0.008 |
| Entamoeba dispar | |  | 14.6 (72) | 15.4 (14) | 0.85 |
| Blastocystis hominis | |  | 4.3 (21) | 3.3 (3) | 1.0 |
| Iodamoeba bütschlii | |  | 3.9 (19) | 1.1 (1) | 0.34 |
| Trichomonas hominis | |  | 2.8 (14) | 6.6 (6) | 0.11 |
| Endolimax nana | |  | 3.5 (17) | 1.1 (1) | 0.33 |
| Chilomastix mesnili | |  | 2.6 (13) | 1.1 (1) | 0.71 |
|  | |  |  |  |  |
| No. of intestinal parasites | |  |  |  |  |
|  | 0 | | 15.2 (75) | 33.0 (30) |  |
|  | 1 | | 33.9 (167) | 29.7 (27) |  |
|  | 2 | | 26.6 (131) | 20.9 (19) |  |
|  | 3 | | 14.6 (72) | 8.8 (8) |  |
|  | ≥ 4 | | 9.6 (47) | 7.7 (7) | 0.002 |

Table shows crude prevalences (unweighted) as percentages and absolute numbers. ^a^, includes samples positive by microscopy only; ^b^, excludes one isolate with mixed assemblage A/B; ^c^, includes 3/16 microscopically detected hookworms without PCR confirmation and species differentiation
